# Influence of Incorporating Recycled Windshield Glass, PVB-Foil, and Rubber Granulates on the Properties of Geopolymer Composites and Concretes

**DOI:** 10.3390/polym15092122

**Published:** 2023-04-29

**Authors:** Van Su Le, Katarzyna Ewa Buczkowska, Roberto Ercoli, Kinga Pławecka, Narcisa Mihaela Marian, Petr Louda

**Affiliations:** 1 Faculty of Mechanical Engineering, Technical University of Liberec, 461 17 Liberec, Czech Republic; katarzyna.ewa.buczkowska@tul.cz (K.E.B.); petr.louda@tul.cz (P.L.); 2 Department of Materials Technology and Production Systems, Faculty of Mechanical Engineering, Lodz University of Technology, 90-001 Lodz, Poland; 3 Department of Pure and Applied Science, University of Urbino, 61029 Urbino, Italy; 4 Faculty of Material Engineering and Physics, Cracow University of Technology, 31-864 Cracow, Poland; k.plawecka96@gmail.com; 5 Department of Chemistry, University of Pavia, 27100 Pavia, Italy; narcisamihaela.marian@unipv.it

**Keywords:** geopolymer, concrete, windshield glass, PVB-Foils, rubber granulate

## Abstract

Waste materials from the automotive industries were re-used as aggregates into metakaolin-based geopolymer (GP), geopolymer mortar (GM), and Bauhaus B20-based concrete composite (C). Specifically, the study evaluates the ability of windshield silica glass (W), PVB-Foils (P), and rubber granulates (G) to impact the mechanical and thermal properties. The addition of the recovered materials into the experimental geopolymers outperformed the commercially available B20. The flexural strength reached values of 7.37 ± 0.51 MPa in concrete with silica glass, 4.06 ± 0.32 in geopolymer malt with PVB-Foils, and 6.99 ± 0.82 MPa in pure geopolymer with rubber granulates; whereas the highest compressive strengths (б_c_) were obtained by the addition of PVB-Foils in pure geopolymer, geopolymer malt, and concrete (43.16 ± 0.31 MPa, 46.22 ± 2.06 MPa, and 27.24 ± 1.28 MPa, respectively). As well PVB-Foils were able to increase the impact strength (б_i_) at 5.15 ± 0.28 J/cm^2^ in pure geopolymer, 5.48 ± 0.41 J/cm^2^ in geopolymer malt, and 3.19 ± 0.14 J/cm^2^ in concrete, furnishing a significant improvement over the reference materials. Moreover, a correlation between density and thermal conductivity (λ) was also obtained to provide the suitability of these materials in applications such as insulation or energy storage. These findings serve as a basis for further research on the use of waste materials in the creation of new, environmentally friendly composites.

## 1. Introduction

The main structural material for construction is concrete, which is primarily made using Portland cement as a binding agent. However, the production of Portland cement is energy-intensive and results in significant greenhouse emissions into the atmosphere, which are much higher than all air transport in the world. It is estimated that 5 to 10 billion cubic meters of concrete and reinforced concrete are produced worldwide each year. The efficiency of the production of this material has a significant impact on people’s lives [1].

In the second half of the 20th century, J. Davidovits introduced the concept of geopolymers, which are made from aluminosilicate mineral raw materials [2]. Their production is five times less CO_2_ compared to Portland cement [3,4,5,6,7]. The advantage of geopolymer technology is the absence of high-temperature synthesis, as well as the possibility of using waste as a precursor or additive. Moreover, properties such as high mechanical strength, thermal and corrosion resistance, and durability [8,9] make them worthy applicative solutions [10,11,12].

Geopolymers are binder systems made from finely dispersed amorphous or crystalline silicate and aluminosilicate materials activated using an alkaline solution [13]. The mechanism behind the production of these inorganic compounds is based on the so-called geopolimerization process. The most common raw materials are kaolin (which is also the most studied precursor), fly ash from coal combustion waste, and blast furnace slags, which are inexpensive and widely available, but alkaline activation involve any material with a high alumina-silicate content [14,15]. 

The process in question first consists of a heat treatment generally of kaolin (K), where the transformation of kaolinite (Kln) into metakaolinite (MKln) takes place at a temperature of between 500 °C and 800 °C. During this process, structural hydroxyl ions are released. Subsequently, the metakaolin (MK) is alkaline activated through an alkaline solution (such as Na_2_SiO_3_, or H_2_O made basic through the addition of known concentrations of NaOH or KOH). By mixing these two components, the dissolution of Si-O, and Al-O occurs, leading to the formation of a supersaturated solution of the two groups. The setting of the material and the resulting condensation (curing or maturation) causes the aggregation and reorganization of the gel into polymers, consisting of alternating silicon and aluminum structures, respectively IV and IV–V coordinated with oxygen. This phase takes place at temperatures between 20 °C and 100 °C in special molds and under controlled conditions for certain and different durations. The chemical composition, solid/liquid ratio, and pH are the main parameters influencing the material’s processability.

The economic and environmental feasibility of using geopolymers is a good incentive to study their industrial implementation. However, geopolymers are not yet broadly used worldwide, mainly due to a lack of research on the topic, the heterogeneity of raw materials, and the lack of regulatory documentation. The composition of materials is not constant; technologies in the construction market are growing and new ideas are emerging that expand the possibilities of their application [16].

In this research, waste such as car scraps was considered, particularly municipal solid waste: (i) windshield, (ii) polyvinyl butyral film from the recycling of old automotive windshields, and (iii) rubber granulate from the recycling process of automotive waste tires. Using these materials as fillers in geopolymer structures is an emerging area of research, promoted also by the European regulation i.e., Directive 2008/98/EC recently updated with 2018/851/UE [17,18]. By default, worn or damaged tires go through a recycling process that yields several secondary materials such as textiles, scrap metal, and rubber granules. To establish a sustainable and efficient geopolymer building materials industry, it is crucial to accumulate and systematically analyze data on the influence of various factors. This will provide the scientific and practical foundations for industrial technologies of geopolymer materials for various construction purposes. Geopolymer composites synthesized from these waste materials represent sustainable construction materials, approaching to reduce the carbon footprint [19,20,21,22].

## 2. Materials and Methods

### 2.1. Raw Materials Used for the Matrix of Geopolymers

The commercial binder used in the study was sourced from Ceské Lupkové Závody, a.s., a company in the Czech Republic. Bausik LK is composed of metakaolin (*ρ* = 1220 kg/m^3^, D90 = 10 μm), and KOH aqueous solution with a pH = 11. It was combined with silica sand (*ρ* = 2650 kg/m^3^, D_90_ = 0.5 mm), a coarse material from the company Sklopisek Strelec, a.s, Hrdonovice, Czech Republic, to enhance the structure of the geomortar and the mechanical properties [23]. The chemical composition of MK and SA is reported in Table 1. 

**Figure 1:**
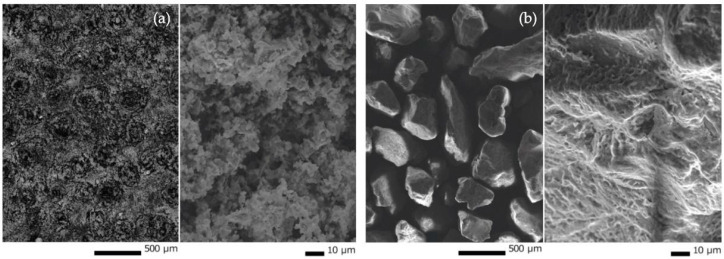
SEM images of (**a**) metakaolin and (**b**) silica sand.

The materials were characterized at the Department of Materials Engineering, Faculty of Materials Engineering and Physics of the Technical University of Krakow using scanning electron microscopy (JEOL, model JSM-IT200), providing detailed information about surface morphology. Figure 1a,b show the structures of metakaolin (MK) and silica sand (SA). The metakaolin exhibits a two-layer crystal structure composed of a silicon-oxygen tetrahedral layer and an alumina octahedral layer. Silica sand, commonly referred to as sharp sand used in construction, contains sharp crystals of quartz, not weathering.

### 2.2. Waste Materials Used as Fillers in the Geopolymers Mortar

**Figure 2:**
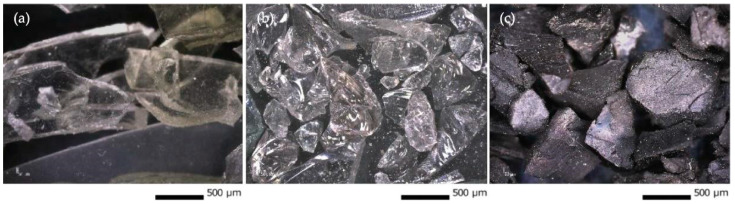
(**a**) Windshield PVB foils—P, (**b**) Windshield Silica Glass—W, and (**c**) Rubber granulates—G processed by ELDAN technology from Denmark.

Three types of municipal solid waste were used as fillers in the Geopolymers Mortar: (i) windshield silica glass (W), (ii) windshield polyvinyl butyral foils (P), and (iii) rubber granulate (G) from the recycling process of car tires (Figure 2a–c).

Silica glass is obtained from the recycling process of car windshields, provided by the company Auto Glass Recycling s.r.o. Only the 1–25 mm fraction was selected for the study without manipulating the material to avoid any energy consumption.

The morphological similarity of the particles to silica sand [24,25,26] which is commonly used as a structuring material in the synthesis of geopolymer, was assumed. This assumption is only correct for particle size at a low magnification. However, at higher magnification, the difference in sharpness of the edges and surface structure can be observed. In the case of waste glass, the edges of the particles are sharper and the surface structure is smoother. These differences can be attributed to the transition of substances from a crystalline state to a glassy state, which is accompanied by a change in the internal structure.

Another type of waste material whose lifespan is often shorter than that of the car itself is the windshield. The windshield must be disposed of in an environmentally friendly manner, so the recycling process comes into play. Essentially, the windshield is composed of glass and a polyvinyl butyral (PVB) film. These components are very strongly bonded. However, the disposal process is more complicated than with traditional waste glass.

The polyvinyl butyral film (PVB) was provided by the company Auto Glass Recycling s.r.o., Šelpice, Slovakia, which deals with several processes, including recycling windshields. Washed PVB fibers with approximately 20–100 mm lengths were selected for the research. Differential scanning calorimetry (DSC) was performed on the material in a nitrogen atmosphere at a temperature from −100 to 250 °C. The influence of temperature on the material starts at 18 °C, whereas 217 and 250 °C represent the limit of bond breaking. During the second heating stage, the material melts at a temperature of 200 °C. 

A detailed Energy Dispersive Spectroscopy EDS analysis is presented in Table 2.

### 2.3. Standard Concrete

The standard concrete was used to compare and demonstrate the feasibility to employ geopolymer as a substitution for cement. The physical properties, compressive strength, and thermal conductivity of B20 concrete as well as durability properties are illustrated in Table 3. Additionally, the microstructural properties of the material were analyzed using scanning electron microscopy spectroscopy.

### 2.4. Methods for the Synthesis of GP, GM, and C

The composites consist of geopolymer precursors or concrete B20, along with aggregates. The pure geopolymer (GP) and the geopolymer malt (GM) were produced by mixing metakaolin as a precursor activated with a potassium hydroxide aqueous solution for 5 min. The ratio between metakaolin and the activator used for the experimentation was fixed at 1:0.9. Instead, the standard concrete (C) was blended for the same time using H_2_O with a ratio of 1:0.1.

The second experimental step, which cast adding (and mixing for 3 min) the above-mentioned waste materials to the GP, GM, and C is processed to investigate their influence as the principal aim of the research. Table 4 illustrates the details of the experimental ratios between such materials.

The hardening treatments of the experimental products were an integral part of the study and were performed with utmost care and precision. The specimens were subjected to heat-drying conditions at room temperature, which was carefully monitored to ensure that the optimal curing conditions were met. The curing process is a crucial step in the development of composites and determines the final properties of the product.

To study the curing rate and the kinetic transformations that take place during the geopolymer hardening process, the specimens were placed in specific molds and allowed to cure for 28 days, the time frame required for the maturation. The molds were designed in such a way that the specimens were uniformly exposed to the curing conditions, thereby ensuring that the results were accurate and reliable.

The curing process plays a crucial role in the development of composites, as it determines the final properties of the products, determining their strength, durability, and resistance to external factors. In conclusion, the hardening treatments of the experimental products were a critical aspect of the study and were carefully carried out to ensure that the specimens reached their maximum potential in terms of their physical properties.

### 2.5. Characterization Methods

The flexural, compression, and impact strength tests, and thermal conductivity analysis were conducted on the geopolymer composites to determine their mechanical and thermal characteristics. These tests were performed using state-of-the-art testing equipment and were carried out following industry standards, to ensure the accuracy and reliability of the results obtained.

The flexural and compression tests were conducted on the specimens of geopolymer composites using an Instron Universal Testing Machine, Model 4202. This machine was equipped with a load cell of 10 kN and a crosshead speed of 2.5 mm/min, which were maintained at ambient temperature during the testing process. The three-point bending tests were performed on specimens with dimensions of (40 × 40 × 160) mm^3^, while the compression tests were performed on cubic specimens of (40 × 40 × 40) mm^3^. To ensure consistent and accurate results, three samples were tested for each series of tests, following the standards outlined in CSN EN 1015-11 [27]. In addition to the flexural and compression tests, impact strength tests were also conducted on each sample. The impact strength was measured using a WPM PS30 device, which measuring range is 0–300 J.

The thermal conductivity was analyzed using the HFM436 Lambda instrument (Netzsch, a.s., Selb, Germany). This analysis was conducted under the standards outlined in ASTM E1225-13 [28]. To obtain accurate thermal conductivity measurements, one sample from each group of dimensions (300 × 300 × 50) mm^3^ was used for testing.

The thermal conductivity has been calculated following the relationship (Equation (1)):

(1) λ=(Q × L)/(A × ΔT) 

where:

λ = Thermal conductivity of the geopolymer composite (W/m·K)

Q = Heat input rate (W)

L = Length of the specimen (m)

A = Cross-sectional area of the specimen (m^2^)

ΔT = Temperature difference across the specimen (K)

The relationship between thermal conductivity and density of geopolymer composites has also been investigated and discussed as result. Generally, as the density of the material increases, its thermal conductivity tends to decrease due to the packing of the aggregate particles becoming tighter, reducing the number of air pockets in the material. Air pockets tend to be poor conductors of heat, so reducing their number tends to increase the thermal conductivity of the material. Other factors such as the composition of the geopolymer binder, the shape and size of the aggregate particles, and the presence of additives also influence this parameter.

## 3. Results and Discussion

Mechanical properties such as flexural, compression, and impact strengths, can be used to determine the ability of a material to resist stress and deformation. Thermal conductivity, on the other hand, provides information about the ability of a material to transfer heat. Table 5 illustrates the results, which are providing valuable information about the behavior of materials under different conditions. These results can be used to evaluate the performance of materials in various applications and to make informed decisions about the suitability of a material for a particular use. This factor is crucial for developing new and improved products and for optimizing the performance of existing materials.

**Figure 3:**
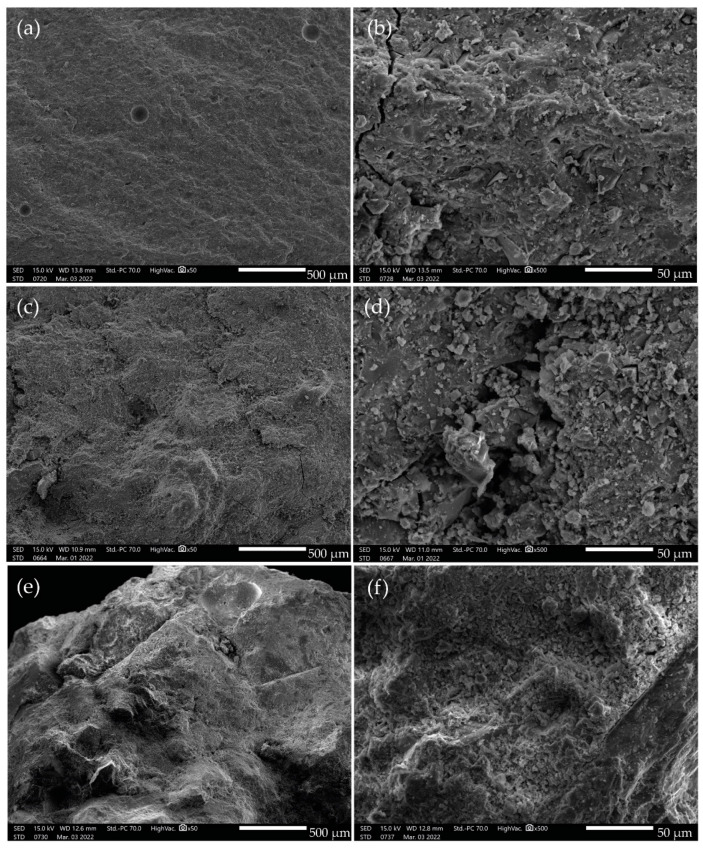
Standards of (**a**,**b**) geopolymer, (**c**,**d**) geopolymer malt, and (**e**,**f**) concrete.

Microstructural and microtextural characteristics of the standards used in the study can be observed in Figure 3a–f. Figure 3a,b show a homogeneous matrix of pure geopolymer, indicating almost complete transformation by alkaline activation of the metakaolin. In contrast, Figure 3c,d illustrate that the addition of silica sand results in a less homogeneous matrix with clasts of varying sizes (<50 µm). The concrete sample Figure 3e,f displays coarser particles and conchoidal cavities, making the product visibly more heterogeneous and complex.

### 3.1. Mechanical Properties

Windshield silica glass (W) does not really change significatively the flexural strength of concrete that outperformed the pure geopolymer and geopolymer malt, which instead undergo a real loss of ***σ***_f_. This loss is reducible to the low reactivity that the waste materials deriving from the windshield glass have in the geopolymers. Generally, the compressive strength with the addition of W decreases for all the specimens, while the impact strength, which follows the same trend, is only improved is C-0.5W with a value of 1.81 ± 0.05 J/cm^2^.

The addition of PVB foils (P) decreases the flexural strengths in all the products but enhances the compressive strength of geopolymer malt and concrete, 1.19 and 1.15 times the standard values of GM and C respectively. Whereas the pure geopolymer decreases its ***σ***_c_ by 20.6%. The impact strength is really improved by PVB foils, especially in geopolymer malt where the value is up to 3.15 times the standard. Concrete is the product less reacting with P improving 1.83 times the ***σ***_i_. The highest compressive strengths are detected in GP-0.1P, GM-0.1P, and C-0.1P (43.16 ± 0.31 MPa, 46.22 ± 2.06 MPa, and 27.24 ± 1.28 MPa) whilst the impact strength (***σ***_i_) in GP-0.3P, and GM-0.3P (5.15 ± 0.28 J/cm^2^, and 5.48 ± 0.41 J/cm^2^).

Rubber Granulate (G) finds its best employment in the pure geopolymer (GP-0.2G) with a flexural strength of 6.99 ± 0.82 MPa. The compressive strengths are reduced in all the materials. On the contrary, the impact strength is improved.

### 3.2. Thermal Conductivity

**Figure 4:**
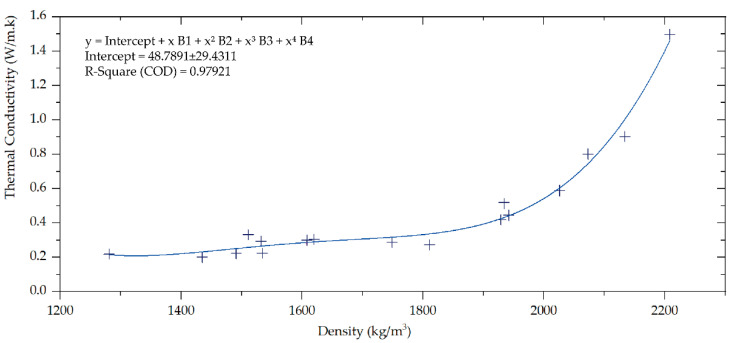
Correlation between thermal conductivity and density.

The density of a material is a key property that can have significant impacts on its performance such as thermal conductivity. It is influenced by several factors, including the curing process and rate of consolidation. These factors can have a significant impact on both the density and thermal conductivity of a material, particularly in the initial stages of reactions. A direct correlation between thermal conductivity and density was established, as shown in Figure 4.

Geopolymers (GPs) exhibit the lowest values in terms of densities and thermal conductivities. However, geopolymer mortars (GMs) and concretes (Cs) seem to have the opposite reaction to these properties. In all cases, increasing the amount of silica glass (W) by 5, 10, or 20 wt.% of metakaolin (MK) causes higher thermal conductivity. On the other hand, the use of PVB foils (P) and rubber granulates (G) is more suitable for achieving low λ. The lowest thermal conductivity of the geopolymer is achieved by adding 30 wt.% MK of PVP-Foils, reaching a value of 0.201 W/m·K in the geopolymer.

## 4. Conclusions

Experimental procedures on the development of composites from the windshield (W), polyvinyl butyral film (P), and rubber granulates (G) have been thoroughly performed and presented in this research. Waste materials used as aggregate into pure geopolymer, geopolymer mortar, and concrete strongly influenced the volume ratio with the main matrix composed of reacted metakaolin, and B20 Cement. Therefore, these ratios have been optimized to ensure crosslinking with the matrix while avoiding crumbling. Their influence on the mechanical and thermal properties of the standards was investigated.

PVB-Foil and Rubber Granulates impact more efficiently in the GP and GM than in the C. The most performing flexural strengths can be observed in GP-0.2G, GM-0.3P, and C-0.5W (6.99 ± 0.82 MPa, 4.06 ± 0.32 MPa, and 7.37 ± 0.51 MPa). When polyvinyl butyral film is added to the GM, the scrap used as an additive material tends to have a good ability to improve its mechanical properties such as the impact strength, with a maximum value of 5.48 ± 0.41 MPa. Geopolymers exhibit the best thermal conductivities compared to concrete, ranging from 0.201 to 0.445 W/m·K. However, geopolymer mortars and concretes show higher values after the addition of W, P, and G.

In conclusion, the results of this study show the promise of using recovered materials from the automotive industry in the geopolymer matrix in terms of physicochemical and mechanical properties.

## Figures and Tables

**Table 1:** Chemical bulk XRF analysis of Metakaolin and Silica Sand.

Oxides (wt.%)								
	SiO_2_	Al_2_O_3_	K_2_O	Fe_2_O_3_	TiO_2_	MgO	CaO	LOI
Metakaolin (MK)	54.10	40.10	0.8	1.10	1.8	0.18	0.13	2.2
Silica Sand (SA)	99.4	-	-	0.04	-	-	-	-

**Table 2:** EDS analyses of the recycled materials.

Element (wt.%)							
Waste	C	O	Si	Al	Ca	Mg	Na
W	-	53.46 ± 0.56	28.23 ± 0.35	10.78 ± 0.23	4.72 ± 0.19	2.33 ± 0.11	0.48 ± 0.06
P	43.5 ± 1.64	43.6 ± 0.99	7.3 ± 0.65	2.47 ± 0.40	1.07 ± 0.05	1.4 ± 0.1	0.57 ± 0.17
G	63.9 ± 3.19	24.9 ± 1.93	0.7 ± 0.08	-	6.9 ± 0.59	-	0.3 ± 0.08

**Table 3:** Technical parameters of the commercial Bauhaus B20 concrete.

B20 Concrete (C)	
Density	2100 kg/m^3^
Grain size	0–4 mm
Compressive strength	Min. 20 MPa
Thermal conductivity	1.50 W/m·K
Consistency	Grade S1–S2
Frost resistance factor	0.75

**Table 4:** Resume of the ratio and nomenclature of the components of Pure Geopolymers, Geopolymer Mortars and Concretes, the waste materials as aggregates.

**Starting Materials**		**GP**	**GM**	**C**
Metakaolin (g)		100	100	-
Alkaline Solution (mL)		90	90	-
Silica Sand (g)		-	100	-
Cement B20 (g)		-	-	100
H_2_O (mL)		-	-	10
**Aggregates**	**m (g)**	**Samples Name**		
Silica Glass (W)	50	GP-0.5W	GM-0.5W	C-0.5W
	100	GP-1W	GM-1W	C-1W
	200	GP-2W	GM-2W	C-2W
PVB-Foils (P)	10	GP-0.1P	GM-0.1P	C-0.1P
	20	GP-0.2P	GM-0.2P	C-0.2P
	30	GP-0.3P	GM-0.3P	C-0.3P
Rubber Granulates (G)	20	GP-0.2G	GM-0.2G	C-0.2G
	60	GP-0.6G	GM-0.6G	C-0.6G

**Table 5:** Density, mechanical, and thermal conductivity results of geopolymers are reported. Values of the standard materials are in bold whereas the blue values indicate the highest mechanical performances and the lowest thermal conductivities for each type (pure geopolymer, geopolymer malt, and concrete).

Sample	ρ (kg/m^3^)	***σ***_f_ (Mpa)	***σ***_c_ (Mpa)	***σ***_i_ (J/cm^2^)	λ (W/m·K)
**GP**	**1608**	**8.97 ± 0.82**	**54.36 ± 2.04**	**1.63 ± 0.05**	**0.301**
GP-0.5W	1749	0.69 ± 0.11	35.90 ± 2.29	1.22 ± 0.12	0.288
GP-1W	1811	0.75 ± 0.17	25.93 ± 1.23	1.26 ± 0.05	0.273
GP-2W	1942	1.41 ± 0.22	15.84 ± 0.91	1.11 ± 0.09	0.445
GP-0.1P	1534	4.20 ± 0.31	**43.16 ± 0.31**	2.70 ± 0.31	0.224
GP-0.2P	1491	3.73 ± 0.76	33.40 ± 0.55	3.78 ± 0.36	0.223
GP-0.3P	1435	2.90 ± 0.29	32.73 ± 0.92	**5.15 ± 0.28**	**0.201**
GP-0.2G	1511	**6.99 ± 0.82**	18.72 ± 0.97	1.85 ± 0.14	0.332
GP-0.6G	1281	4.94 ± 1.05	9.67 ± 0.51	1.80 ± 0.17	0.219
**GM**	**1808**	**4.54 ± 0.81**	**38.83 ± 0.67**	**1.81 ± 0.05**	**0.997**
GM-0.5W	1929	2.91 ± 0.60	29.90 ± 1.07	1.48 ± 0.05	0.419
GM-1W	2026	2.01 ± 0.13	23.35 ± 1.10	1.52 ± 0.05	0.588
GM-2W	2134	2.31 ± 0.13	19.83 ± 0.35	1.67 ± 0.09	0.903
GM-0.1P	1685	3.26 ± 0.23	**46.22 ± 2.06**	4.00 ± 0.57	0.551
GM-0.2P	1620	3.66 ± 0.45	40.54 ± 1.45	4.81 ± 0.14	0.305
GM-0.3P	1532	**4.06 ± 0.32**	36.74 ± 1.63	**5.48 ± 0.41**	0.293
GM-0.2G	1683	3.78 ± 0.23	30.78 ± 1.31	4.00 ± 0.57	0.53
GM-0.6G	1464	2.81 ± 0.34	8.76 ± 0.33	4.81 ± 0.14	**0.266**
**C**	**2208**	**7.47 ± 0.71**	**23.54 ± 0.46**	**1.74 ± 0.10**	**1.496**
C-0.5W	2073	7.37 ± 0.51	18.99 ± 0.93	1.81 ± 0.05	0.8
C-1W	1934	7.24 ± 0.34	8.36 ± 0.87	1.48 ± 0.14	0.519
C-2W	2148	7.35 ± 0.48	8.24 ± 0.75	1.58 ± 0.12	0.853
C-0.1P	2257	5.50 ± 0.93	**27.24 ± 1.28**	2.74 ± 0.14	1.330
C-0.2P	2243	3.09 ± 0.37	22.95 ± 1.65	**3.19 ± 0.14**	0.626
C-0.3P	2103	3.53 ± 0.23	21 ± 1.23	3.03 ± 0.12	0.478
C-0.2G	1903	3.87 ± 0.25	8.05 ± 0.83	1.81 ± 0.05	0.801
C-0.6G	1518	0.93 ± 0.05	1.56 ± 0.09	1.52 ± 0.05	**0.502**

Note: GP-W = geopolymers with windshield silica glass; GP-P = geopolymers with windshield PVB foils; GP-G = geopolymers with rubber granulates; GM-W = geopolymer mortar with windshield silica glass; GM-P = geopolymer mortar with PVB foils; GM-G = geopolymer mortar with rubber granulates; C-W = concrete with windshield silica glass; C-P = concrete with PVB foils.

## Data Availability

Not applicable.

## References

[1] Watts J. (2019). Concrete: The Most Destructive Material on Earth. Guardian.

[2] Davidovits J. (1989). Geopolymers and Geopolymeric Materials. J. Therm. Anal..

[3] Davidovits J. (1994). Global Warming Impact on the Cement and Aggregates Industries. World Resour. Rev..

[4] Davidovits J. (2005). Geopolymer, Green Chemistry and Sustainable Development Solutions. Proceedings of the World Congress Geopolymer 2005.

[5] Le V.S., Szczypinski M.M., Hájková P., Kovacic V., Bakalova T., Volesky L., Hiep L.C., Louda P. (2020). Mechanical Properties of Geopolymer Foam at High Temperature. Sci. Eng. Compos. Mater..

[6] Le V.S., Hájková P., Kovačič V., Bakalova T., Voleský L., Le C.H., Seifert K.C., Peres A.P., Louda P. (2019). Thermal Conductivity of Reinforced Geopolymer Foams. Ceram.-Silikáty.

[7] García-Segura T., Yepes V., Alcalá J. (2014). Life Cycle Greenhouse Gas Emissions of Blended Cement Concrete Including Carbonation and Durability. Int. J. Life Cycle Assess..

[8] Davidovits J., Sawyer J.L. (1984). Early High-Strength Mineral Polymer 1985. US Patent.

[9] Kearsley E.P., Wainwright P.J. (2001). The Effect of High Fly Ash Content on the Compressive Strength of Foamed Concrete. Cem. Concr. Res..

[10] Davidovits J. (1991). Geopolymers: Inorganic Polymeric New Materials. J. Anal. Calorim..

[11] Nguyen V.V., Le V.S., Louda P., Szczypiński M.M., Ercoli R., Růžek V., Łoś P., Prałat K., Plaskota P., Pacyniak T. (2022). Low-Density Geopolymer Composites for the Construction Industry. Polymer.

[12] Ercoli R., Laskowska D., Nguyen V.V., Le V.S., Louda P., Łoś P., Ciemnicka J., Prałat K., Renzulli A., Paris E. (2022). Mechanical and Thermal Properties of Geopolymer Foams (GFs) Doped with by-Products of the Secondary Aluminum Industry. Polymer.

[13] BAUCIS LK: ČLUZ. http://www.cluz.cz/en/baucis-lk.

[14] Aygörmez Y. (2021). Evaluation of the Red Mud and Quartz Sand on Reinforced Metazeolite-Based Geopolymer Composites. J. Build. Eng..

[15] Şahin F., Uysal M., Canpolat O. (2021). Systematic Evaluation of the Aggregate Types and Properties on Metakaolin Based Geopolymer Composites. Constr. Build. Mater..

[16] Kohout J., Koutník P. (2020). Effect of Filler Type on the Thermo-Mechanical Properties of Metakaolinite-Based Geopolymer Composites. Materials.

[17] European Parliament and of the Council (2008). Directive 2008/98/EC of the European Parliament and of the Council.

[18] European Parliament (2018). Regulation (EU) 2018/848 of the European Parliament and of the Council of 30 May 2018.

[19] Pawlik T., Michalik D., Sopicka-Lizer M., Godzierz M. (2017). Manufacturing of Light Weight Aggregates from the Local Waste Materials for Application in the Building Concrete. Trans. Technol. Publ..

[20] Rigotti D., Dorigato A. (2022). Novel Uses of Recycled Rubber in Civil Applications. Adv. Ind. Eng. Polym. Res..

[21] Arabi N., Meftah H., Amara H., Kebaïli O., Berredjem L. (2019). Valorization of Recycled Materials in Development of Self-Compacting Concrete: Mixing Recycled Concrete Aggregates–Windshield Waste Glass Aggregates. Constr. Build. Mater..

[22] Khouri S., Behun M., Knapcikova L., Behunova A., Sofranko M., Rosova A. (2020). Characterization of Customized Encapsulant Polyvinyl Butyral Used in the Solar Industry and Its Impact on the Environment. Energies.

[23] Nuaklong P., Sata V., Chindaprasirt P. (2016). Influence of Recycled Aggregate on Fly Ash Geopolymer Concrete Properties. J. Clean. Prod..

[24] Zhou W., Shi X., Lu X., Qi C., Luan B., Liu F. (2020). The Mechanical and Microstructural Properties of Refuse Mudstone-GGBS-Red Mud Based Geopolymer Composites Made with Sand. Constr. Build. Mater..

[25] Nematollahi B., Ranade R., Sanjayan J., Ramakrishnan S. (2017). Thermal and Mechanical Properties of Sustainable Lightweight Strain Hardening Geopolymer Composites. Arch. Civ. Mech. Eng..

[26] Zhang G., He J., Gambrell R.P. (2010). Synthesis, Characterization, and Mechanical Properties of Red Mud–Based Geopolymers. Transp. Res. Rec..

[27] ČSN EN 1015-11 (722400)—Zkušební Metody Malt pro Zdivo—Část 11: Stanovení Pevnosti Zatvrdlých Malt v Tahu za Ohybu a v Tlaku–Duben 2020—Technické Normy—Ing. Jiří Hrazdil. https://shop.normy.biz/detail/509887.

[28] Standard Test Method for Thermal Conductivity of Solids Using the Guarded-Comparative-Longitudinal Heat Flow Technique. https://www.astm.org/e1225-13.html.

